# The Inactivated *gE*/*TK* Gene*-*Deleted Vaccine Against Pseudorabies Virus Type II Confers Effective Protection in Mice and Pigs

**DOI:** 10.3389/fmicb.2022.943707

**Published:** 2022-08-04

**Authors:** Yu-Lan Jin, Di Yin, Gang Xing, Yan-Ming Huang, Chun-Mei Fan, Cheng-Fei Fan, Xiao-Huo Qiu, Wei-Ren Dong, Yan Yan, Jin-Yan Gu, Ji-Yong Zhou

**Affiliations:** ^1^Ministry of Agriculture (MOA) Key Laboratory of Animal Virology, Center for Veterinary Sciences, Zhejiang University, Hangzhou, China; ^2^The Experimental Teaching Center, College of Animal Sciences, Zhejiang University, Hangzhou, China; ^3^State Key Laboratory for Diagnosis and Treatment of Infectious Diseases, First Affiliated Hospital, Zhejiang University, Hangzhou, China

**Keywords:** Pseudorabies virus type II, gE/TK deletion, inactivated vaccine, safe, protection

## Abstract

The highly virulent and antigenic variant of Pseudorabies virus (PRV) that emerged from classical Bartha-K61-vaccinated pig herds has caused substantial economic losses to the swine industry in China since 2011. A safe and more effective vaccine is most desirable. In this study, a gE/TK gene-deficient PRV, namely, HD/c, was constructed based on a PRV type II DX strain isolated from a commercial vaccine-immunized farm and the HD/c-based inactivated vaccine was formulated and evaluated for its safety, immunogenicity, and protective efficacy in mice and piglets. The resulting PRV HD/c strain has a similar growth curve to the parental DX strain. After vaccination, the inactivated HD/c vaccine did not cause any visible gross pathological or histopathological changes in the tissues of mice and piglets and provided rapid and potent protection against the challenge of the classical and variant PRVs at day 21 post-vaccination in mice. A single immunization of 10^8.5^TCID_50_ inactivated PRV HD/c strain-elicited robust immunity with high titer of neutralizing antibody and provided complete protection from the lethal challenge of PRV DX strain in piglets. These results indicated that the inactivated PRV HD/c vaccine with the deletion of *gE*/*TK* genes was a safe and effective PRV vaccine candidate for the control of PRV.

## Introduction

Pseudorabies (PR), also known as Aujeszky's disease, is caused by pseudorabies virus (PRV), a member of the family *Herpesviridae*, subfamily *Alphaherpesvirinae* and genus Varicellovirus (Hanson, 1954). PRV can infect numerous mammals such as mice, pigs, sheep, minks, and dogs, but the family *Suidae* is the only natural reservoir for the virus (Pomeranz et al., 2005; Mettenleiter, 2008; Fonseca et al., 2010; Müller et al., 2011; Boadella et al., 2012). The infected pigs are often manifested by serious respiratory tract symptoms, and neurological phenotypes, with a high-mortality rate. PRV can also lead to latent infection and reproductive failures including spontaneous abortion, fetal malformation and fetal mummies in pregnant sows, causing huge economic losses to the swine industry in many countries (Pomeranz et al., 2005). PRV is a double-stranded DNA virus, with a genome of around 150 kb that consists of a unique short region (US, 9 kb), a unique long region (UL, 110 kb), a terminal repeat sequence (TRS), and an internal repeat sequence (IRS) (van Oirschot et al., 1996). PRV encodes at least 11 different glycoproteins (gB, gC, gD, gE, gG, gH, gI, gK, gL, gM, and gN) (Dong et al., 2014), which are classified as essential and non-essential based on their requirement for viral replication (van Oirschot et al., 1996; Klupp et al., 2004). The glycoprotein gE is encoded by the US8 gene, which is associated with cell fusion, virus diffusion between cells, neurotropism, and virion release. TK was coded by UL23, one of virulence genes, and its deletion led to decrease virulence evidently (Pomeranz et al., 2005). As a viral enzyme, TK mediates the virus replication and spread of PRV in the central nervous system (Ferrari et al., 1998). Deletion of *gE* and *TK* genes can reduce the neurotoxicity of the virus without affecting its immunogenicity (Mettenleiter et al., 1994). Moreover, *gE*, as a marker gene, is used to distinguish natural infection from vaccination when the vaccine lacks the *gE* gene (Wang et al., 2018).

Due to strict implementation of national eradication programs and vaccination with traditional PRV vaccine, PR has been eradicated from domesticated pigs in North America and a number of European countries (Müller et al., 2011). In China, PRV live-attenuated vaccine such as Bartha-K61 has been widely used to effectively control PR during the past several decades (Yuan et al., 1983). However, since late 2011, newly-emerged PRV variants (PRV genotype II) have led to the outbreaks of PR in many pig herds that had been vaccinated with the Bartha-K61 vaccine (An et al., 2013; Wang et al., 2014; Yu et al., 2014; Wang Y. et al., 2015; Ye et al., 2015; Sun et al., 2016), indicating that the Bartha-K61 vaccine failed to protect piglets from the emerging PRV variants (Cong et al., 2016). Thus, a safe and more effective vaccine is urgently needed for the control of PRV variants. Recently, various classical PRV vaccines have been developed, such as those vaccines with the gene deletion of *gE, gI*/*gE, gE*/*TK, gI*/*gE*/*TK*, and *gI*/*gE*/*TK*/*UL13*, respectively (Gu et al., 2015; Wang T. et al., 2015; Cong et al., 2016; Wang et al., 2018; Li et al., 2021; Lv et al., 2021). However, the safety or immunogenicity of these live-attenuated vaccines may be far from being satisfactory (Li et al., 2021).

Given that the use of PRV live-attenuated vaccine may lead to the emergence of novel PRV variants (Li et al., 2020), accordingly, the inactivated vaccines might be the most promising candidate for the development of a safer and more effective PRV vaccine. In the present study, we isolated and identified a PRV II variant, named PRV-DX, from a Bartha-K61 vaccine immunized pig farm. Furthermore, a *gE*/*TK* gene-deficient PRV (named HD/c strain) was constructed, and an inactivated vaccine against PRV was developed and evaluated for its safety and protective efficacy against both classical PRV and the emerging variants. Our results showed that this PRV vaccine was safe and provided effective protection against the classical strain SC and emerging strain DX. Our study indicated that the inactivated PRV HD/c vaccine with the deletion of *gE*/*TK* genes might be a safe and potent PRV vaccine candidate for controlling the PRV in China.

## Materials and Methods

### Cells and Viruses

The Vero and porcine kidney (PK-15) cells were routinely cultured in a high-glucose Dulbecco modified Eagle's medium (DMEM; Invitrogen, Carlsbad, CA) with 10% fetal bovine serum (Gibco, Carlsbad, CA, USA) at 37°C with 5% CO_2_ in a humidified incubator. All the cell lines tested negative for mycoplasma. The PRV DX strain (PRV genotype II) was isolated in 2012 from the brain samples of a diseased piglet collected in a farm of Zhejiang Province, China, and was preserved by Key Laboratory of Animal Virology of Ministry of Agriculture, Zhejiang University. PRV strain SC, a classical PRV strain was kindly provided by UBEN animal vaccines company, Ltd. Hangzhou, China.

### PRV DX Whole Genome Sequencing and Phylogenetic Analysis

Viral genome DNA of PRV DX strain was extracted as described previously (Szpara et al., 2011), and PacBio's SMRT (single molecule real-time) sequencing, third-generation sequencing technology requires no PCR amplification and the read length is 100 times longer than that of NGS, was utilized for the whole genome sequencing by BGI Group (Beijing Genomics Institute, China). The ML tree was build using RAxML–NG (v1.1.0) (Minh et al., 2020), the best model GTR+F+R3 was chosen among 286 DNA models by ModelFinder of IQ-TREE (v1.6.12) (Kozlov et al., 2019), according to Bayesian Information Criterion. The autoMRE bootstrapping convergence criterion was applied to determine the most suitable number of replicates instead of the default 1,000 replicates. Bootstrapping convergence was considered to be reached if over 99% permutations have low WRF distances (< 0.03). The unrooted tree was visualized by iTOL (v6.5.2).

### Generation of Recombinant Virus and Plaque Assay

PRV DX viral DNA was extracted as mentioned earlier and the transfer plasmid pUC18-gE-loxp-cherry (3 μg) was co-transfected with the genomic DNA of PRV DX (1 μg) into Vero cells for homologous recombination. After six times of plaque purification, the gE-deleted virus expressing cherry was generated and named as PRV DX-gE-loxp-cherry. Afterward, the genomic DNA of PRV DX-ΔgE-cherry was extracted and dealt with cre recombinase (New England Biolabs, USA), then the viral DNA was extracted and transfected into Vero cells. Undergoing plaque purification for six times, PRV gE-deleted virus was generated and named as PRV DXΔgE strain. Then 3 μg of pUC18-TK-loxp-EGFP plasmid and 1 μg of PRV DXΔgE genome DNA were co-transfected into Vero cells. Plaques with green fluorescence were purified by six rounds, viral DNA was extracted and excised with cre recombinase and then transfected into Vero cells as described earlier. After another plaque purification for six times, the gE/TK-deleted PRV (namely, HD/c) was generated, and the genome were confirmed by sequencing (Biosune, China).

### One-Step Growth Kinetics and Genetic Stability of PRV HD/c Strain

The virus was propagated in Vero cells, and the viral titer of the culture was determined by the Reed–Muench method (Reed and Muench, 1938). Vero cells in 12-well cell plates (90% confluent) were inoculated with the PRV DX or HD/c strain at an MOI of 10 and incubated at 37°C for 1 h. Then the cell monolayers were washed three times with phosphate buffered solution (PBS). Thereafter, the inoculum was replaced with fresh medium, and the cell culture was harvested at 4, 8, 12, 18, 24, 30, 36, 48, 60, and 72 h post infection (hpi). After two freeze-thaw cycles, the cellular supernatant was tested on Vero cells and the virus titers showed as 50% tissue culture infectious dose (TCID_50_).

To test the genetic stability of PRV HD/c strain, recombinant virus was passaged on Vero cells for 30 rounds, followed by extracting the viral DNA. Gene-specific primers, TK623-F/R and gE332-F/R (listed in Table 1), were used to amplify the PRV UL23 and US8 gene, respectively. The PCR amplicons of gE and TK genes were further identified by DNA sequencing.

**Table 1:** List of primers used in this study.

**Primers**	**Sequence (5'-3')**	**Restrict enzyme sites**	**Length (bp)**
F-LgE	CGGAATTCGGACACCGACGAGCTAAAAGC	EcoR I	1,702
R-LgE	GGGGTACCTGGCAGGCGGTCTCGAAGCAC	Kpn I	
F-RgE	GGGGATCCGGGCGGCTGTTTGTGCTGGCG	BamH I	1,369
R-RgE	CCCAAGCTTCGTGCGTCTCGGTGGTGATGTAG	Hind III	
F-Mcherry	TACAAGTCCGGACTCGGATCTAGATAACTG	-	4,722
R-Mcherry	CAGTTATCTAGATCCGAGTCCGGACTTGTA	-	
F-McherryCPS	GGGGTACCATAACTTCGTATAATGTATGCTATACGAAGTTATTAGTTATTAATAGTAATCAA	Kpn I	1,704
R-McherryCPS	CGGGATCCATAACTTCGTATAGCATACATTATACGAAGTTATGATGAGTTTGGACAAACCAC	BamH I	
F-LTK	CGGAATTCATCCTCCGGATCTACCTC	EcoR I	455
R-LTK	GGGGTACCAGCGAGGCCACCACCAGG	Kpn I	
F-RTK	CGGGATCCATGGACGCGCTCGTGGCC	BamH I	527
R-RTK	CCCAAGCTTAGCTGGAAGACGAACCAC	Hind III	
F-EGFP	GGGGTACCAATAGTAATCAATTACGGGGTCATT	-	4,700
R-EGFP	CGGGATCCAGATACATTGATGAGTTT	-	
F-EGFPCPS	GGGGTACCATAACTTCGTATAATGTATGCTATACGAAGTTAT AATAGTAATCAATTACGGGGTCATT	Kpn I	1,705
R-EGFPCPS	CGGGATCCATAACTTCGTATAGCATACATTATACGAAGTTATAGATACATTGATGAGTTT	BamH I	
TK623-F	GTTCGTAGAAGCGGTTGTGGCA	-	623
TK623-R	CGTGTTGACCAGCATGGCGTAG	-	
gE332-F	GCTCTGCGTGCTGTGCTCC	-	332
gE332-R	TCGTCACTTCCGGTTTCTCC	-	

### Animal Experiments

#### Inactivated Vaccine Preparation and Test

Different titer of PRV HD/c supernatant (10^6.3^, 10^6.8^, 10^7.0^, 10^7.3^, 10^7.5^, 10^7.8^, 10^8.0^, and 10^8.5^ TCID_50_/ml) was inactivated by β-propiolactone (1:1,000) at 4°C for 48 h, followed by incubating the inactivated virus at 37°C for 2 h to hydrolyze excess β-propiolactone. Vero cells in 6-well cell plates were inoculated with the inactivated virus at 37°C for 2 h. Then, the cell monolayers were washed three times with PBS, and the supernatant was replaced with fresh medium. Cytopathic effect (CPE) was detected after the third virus blind passage. The completely inactivated virus was homogenized with the Montanide^TM^ ISA 28VG (SEPPIC, France), an adjuvant with W/O/W emulsion.

#### Immunologic Safety Assay in Mice and Piglet Models

For the sake of safety analysis, fifteen 6-week-old SPF BALB/c mice randomly assigned to 3 groups. Group 1 was inoculated intraperitoneally with a dose of 10^7.5^ TCID_50_/0.1 ml (0.4 ml/mouse) inactivated PRV HD/c. Group 2 was inoculated intraperitoneally with DMEM. For comparison, group 3 did not receive any administration as MOCK group. The body weight of each mouse was weighed from day 0 post-immunized (dpi) to 7 dpi. All the mice were euthanatized and humanely necropsied at 7 dpi. The absorption of inactivated vaccine and pathological changes in vaccinated mice was observed.

The safety of inactivated PRV HD/c was also assessed in 14-day-old piglets. In total, ten 2-week-old piglets free of PRRSV, PCV2, CSFV, and PRV antibodies were randomly divided into 2 groups. Group 1 was inoculated intramuscularly with a dose of 10^8.5^ TCID_50_/ml (4 ml/pig) inactivated PRV HD/c. Group 2 served as non-treated control. All the piglets were euthanized and necropsied at 14 dpi, and the pathological and histopathological changes were observed.

#### Protective Assay in Mice

In total, one hundred and fifty 6-week-old specific-pathogen-free (SPF) BALB/c mice randomly assigned to 15 groups, ten mice per group. Every group inoculated intraperitoneally (i.p.) with different dose of the inactivated PRV HD/c vaccine (10^7.8^, 10^7.3^, 10^6.8^, and 10^6.3^ TCID_50_), respectively, three groups for each dose. DMEM was given to the mock-immunized group through the same route. Each group was challenged with lethal dose of PRV DX strain (10^5.6^ TCID_50_) at 21, 28, or 35 dpi, respectively. To validate whether the inactivated PRV HD/c vaccine could induce cross-immune responses and cross-protection against PRV genotype I, forty 6-week-old SPF BALB/c mice randomly assigned to 4 groups, 10 mice per group. In two Groups were inoculated intraperitoneally with inactivated PRV HD/c (10^7.8^ TCID_50_), followed by challenging with PRV variant DX strain (10^5.6^ TCID_50_) and classical PRV SC strain (10^5.6^ TCID_50_), respectively. Another two groups were directly challenged with PRV variant DX strain (10^5.6^ TCID_50_) and classical PRV SC strain (10^5.6^ TCID_50_) as control. The survival rates of each group were calculated until 14 days post challenge (dpc). Disease progression of mice was monitored by weight loss and deaths.

#### Protective Test in Piglets

In total, twenty-five 2-week-old piglets which are free of PRRSV, PCV2, CSFV, and PRV antibodies were randomly divided into five groups. In total, four groups were inoculated intramuscularly with different dose (10^8.5^, 10^8.0^, 10^7.5^, and 10^7.0^ TCID_50_) of the inactivated PRV HD/c vaccine. DMEM was given to the mock-inoculated piglets. Blood samples were collected at 0, 7, 14, 21, 28, and 35 dpi for testing the gE- and gB-specific antibodies by enzyme-linked immunosorbent assay (ELISA). All the piglets were challenged with PRV DX at a dose of 10^5.4^ TCID_50_ at 21 dpi. Clinical signs and rectal temperatures were collected from 0 to 14 dpc. All the surviving piglets were euthanatized and humanely necropsied, and the brain, lung, liver, and tonsil were collected for pathological examination by histology.

#### Evaluation of Immune Efficacy of PRV HD/c Vaccine on Pregnant Sows

Five sows free of CSFV, PRRSV, PCV2, and PRV antibodies were set up for timed mating, and at gestation day 7, three pregnant sows were vaccinated *via* intramuscular injection using 10^8.5^ TCID_50_ inactivated PRV HD/c vaccine, two other sows were inoculated DMEM (2 ml). In total, 3 weeks after the first immunization, the pregnant sows were inoculated with inactivated PRV HD/c or DMEM again. All the newborn piglets were blood sampled at 4 weeks of age. In total, five piglets were farrowed by vaccinated sow named group A, and five piglets were farrowed by non-vaccinated sow named group B. Groups A and B (4 weeks of age) were challenged with 10^5.4^ TCID_50_ PRV DX by nasal drip. Clinical signs were monitored from 0 to 14 dpc. All th surviving piglets were euthanatized and humanely necropsied at 15 dpc while piglets that died during animal experiments were necropsied immediately.

### Serological Tests

Pseudorabies virus-specific gE and gB antibodies were detected by commercially available ELISA kits (IDEXX Laboratories, Westbrook, ME, USA) according to the manufacturer's directions. The neutralizing antibody against PRV was tested as described previously with some modifications (Yu et al., 2016; He et al., 2019). The titers of neutralizing antibody were calculated from no CPE according to the Reed–Muench formula.

### Statistical Analysis

Data were analyzed using the SPSS 14.0 software. All the data are presented as mean ± SE. The differences in neutralizing antibody and virus titers among the different groups were measured by one-way repeated measurement ANOVA and least significance difference (LSD). Differences were considered statistically significant at *p* < 0.05.

## Result

### PRV DX Whole Genome Sequencing and Phylogenetic Analysis

The complete genome sequence of the PRV DX strain was obtained by PacBio reads, and the assembly of the scaffolds resulted in a genome size of 143,754 bp (GenBank Accession no. MZ063026). The G+C content of the PRV DX genome was 73.59% and a total of 69 open reading frames (ORFs) were predicted. Moreover, 96 genome sequences of PRV were downloaded from the NCBI database, followed by constructing a rootless phylogenetic tree. Results exhibited that PRV strains were clearly clustered into two big branches including the PRV genotype I and genotype II, and the isolated PRV–DX clustered with other emerging PRV strains since 2011 in China and was classified into the PRV genotype II branch (Supplementary Figure 1).

### Generation of Recombinant gE/TK-Deleted Virus

**Figure 1:**
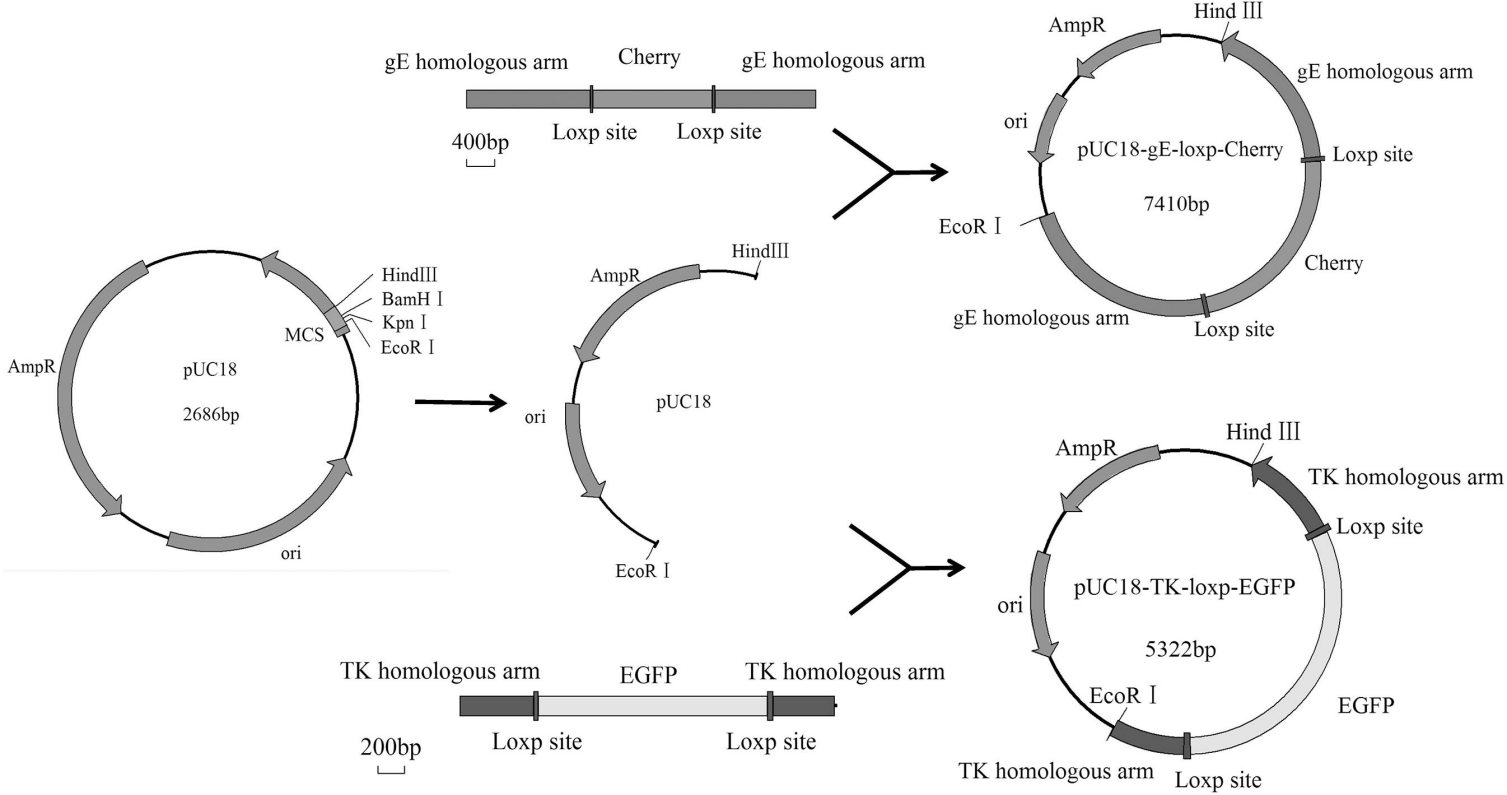
Schematic diagrams of recombinant plasmids.

**Figure 2:**
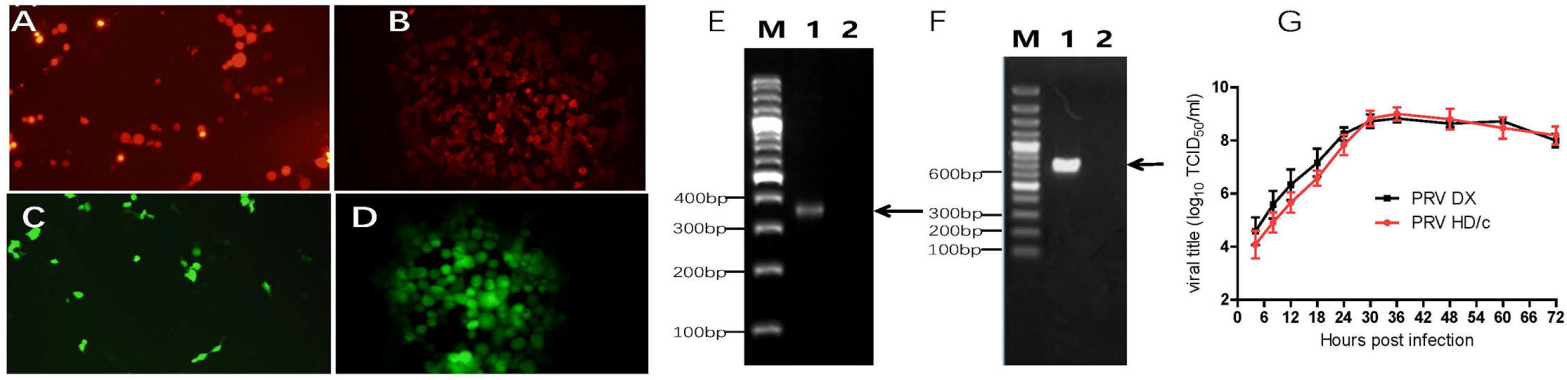
Identification of recombinant PRV. **(A)** Red fluorescence on Vero cells after the cotransfection of pUC18-gE-loxp-Cherry and PRV DX viral DNA. **(B)** Vero cells infected with PRV DX gE^−^/Cherry+. **(C)** Green fluorescence on Vero cells after the cotransfection of PRV DX gE-viral DNA and pUC18-TK-loxp-EGFP. **(D)** Green fluorescence on Vero cells infected with PRV DX gE^−^/TK^−^/EGFP+. **(E)** PCR analysis of recombinant PRV (p30) with primers gE332; Lane 1: PRV DX; Lane 2: PRV HD/c; M: DNA ladder 3000. **(F)** PCR analysis of recombinant PRV (p30) with primers TK623; M: DNA ladder 3000; Lane 1: PRV DX; Lane 2: PRV HD/c. **(G)** One-step growth curves of the PRV DX and PRV HD/c. Vero cells at high confluence (>90%) were infected (MOI = 10) with the PRV DX or PRV HD/c. The cell cultures were harvested at the indicated time points (4, 8, 12, 18, 24, 30, 36, 48, 60, and 72 h), and titers were determined on the Vero cells. The mean values with standard error of mean are shown.

The Cre/Loxp system was utilized to delete the *TK* and *gE* genes from the genome of PRV DX strain. The transfer vectors of pUC18-gE-loxp-Cherry and pUC18-TK-loxp-EGFP were constructed (Figure 1). After co-transfection of Vero cells with PRV DX viral DNA and pUC18-gE-loxp-Cherry, red fluorescence was observed by fluorescence microscopy (Figure 2A). After 6 rounds of plaque purification (Figure 2B), the genome of PRV DX-gE-loxp-cherry was collected. By further plaque purification, PRV DXΔgE strain was obtained and verified. The DNA of PRV DXΔgE and pUC18-TK-loxp-EGFP vector were co-transfected into Vero cells (Figure 2C), EGFP positive plaque was purified (Figure 2D). The DNA of PRV DXΔgEΔTK-loxp-EGFP was extracted and treated with cre recombinase to delete EGFP cassette. At last, the gE/TK-defected recombinant virus was generated and further confirmed by PCR with primers gE332 and TK632 (Figures 2E,F). By passaging it on Vero cells for 30 passages and genome sequencing, recombinant gE/TK-deleted PRV HD/c strain was shown to be stable. One-step growth curve showed that there was no significant difference in the replication kinetics between the parental PRV DX strain and the recombinant HD/c strain (Figure 2G). No significant difference in viral titers was observed and both strains reached the peak in titer (10^9.25^ TCID_50_/ml) at 30 h post infection (hpi). These data suggested that the genes gE/TK had been knocked out in PRV strain DX and the HD/c virus had similar replication ability with wild-type PRV strain DX.

### Safety of Inactivated PRV HD/c Vaccine in Mouse and Piglet

**Figure 3:**
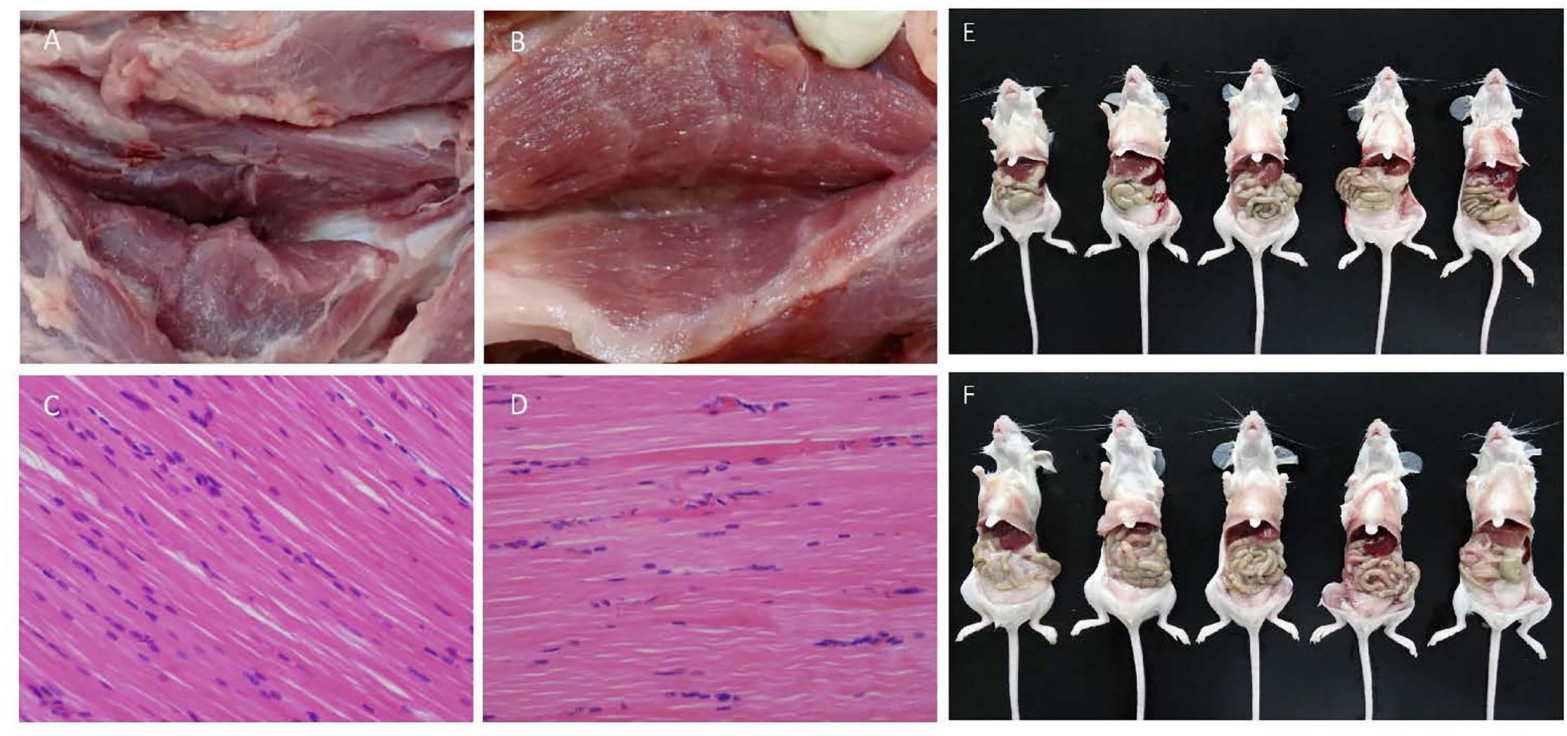
Immunologic safety assay. **(A,B)** Pathological changes of injection site of double-dose (0.4 ml) inactivated PRV HD/c and DMEM immunized piglets at 14 dpi, respectively. **(C,D)** Histopathological observation of inactivated PRV HD/c and DMEM immunized piglets, respectively. Sections were stained with hematoxylin and eosin (HE) and photographed at 100 × magnification. **(E,F)** Pathological changes of mice immunized with double-dose inactivated PRV HD/c and untreated group at 7 dpi, respectively.

The inactivation effect of β-propiolactone was evaluated by blind passage in Vero cell for three rounds. No CPE was observed during the passaging. After injection of the inactivated vaccine, no obvious stress response was observed in the vaccinated mice and piglets. There were no significant differences in pathological and histopathological alterations between the immunized and the non-immunized group in piglet models (Figures 3A–D). Also, there were no macroscopic residues in enterocoelia of vaccinated mice at 7 dpi (Figures 3E,F). These data showed that the β-propiolactone inactivated HD/c antigen is safety to pigs and mice.

### The Inactivated PRV HD/c Vaccine Provide Effective Protection Against the Classical and Emerging PRV in Mice

**Figure 4:**
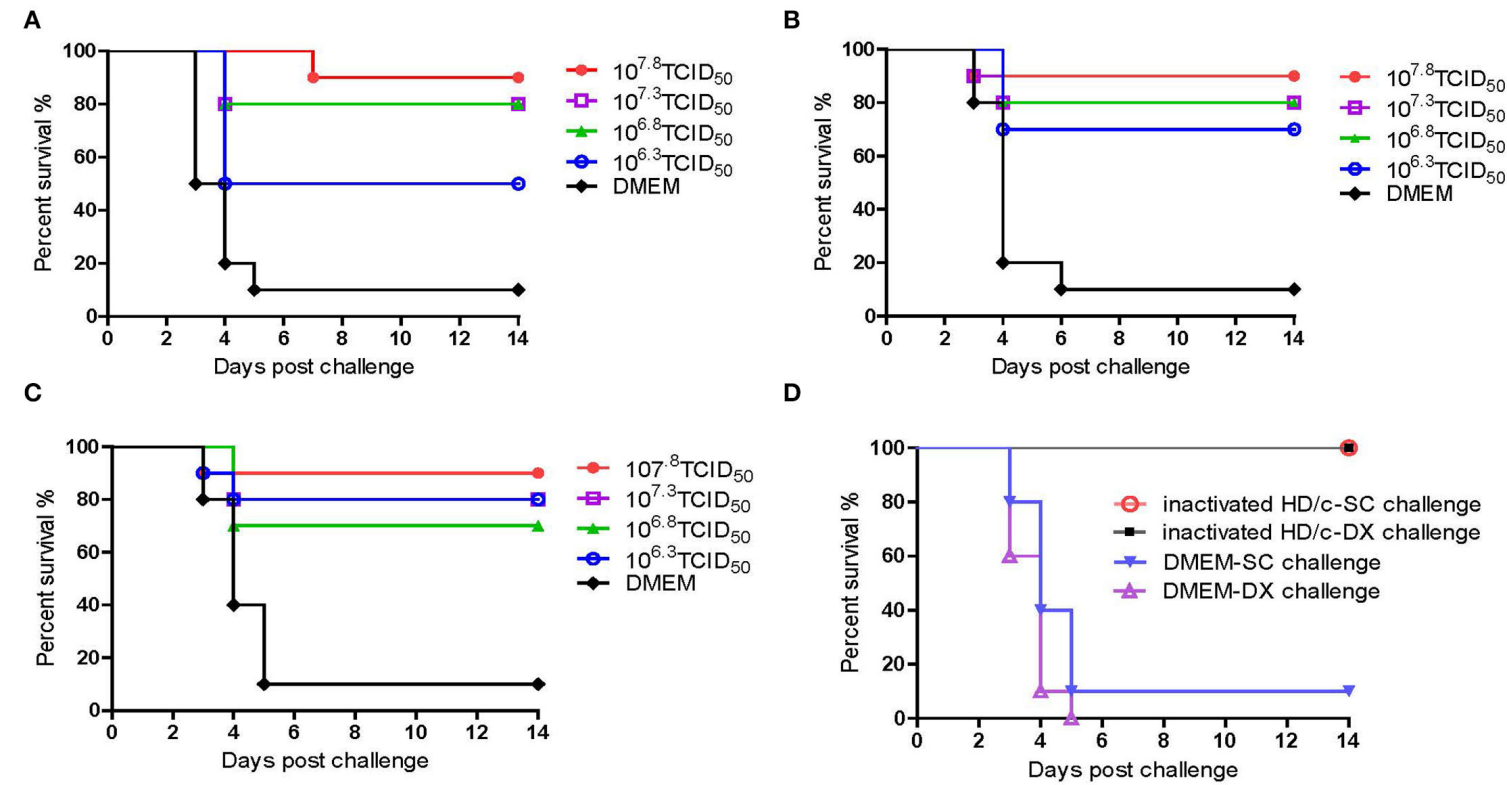
Protective efficacy of inactivated PRV HD/c immunization in mouse model. Survival rates of mice (*n* = 10) immunized with different dose inactivated PRV HD/c (10^6.3^, 10^6.8^, 10^7.3^, and 10^7.8^ TCID_50_) challenge with wide type PRV DX strain (10^5.6^TCID_50_) at 21 dpi **(A)**, 28 dpi **(B)**, and 35 dpi **(C)**. **(D)** Survival rates of inoculated mice (*n* = 10) challenged with PRV DX or SC (10^5.6^ TCID_50_) at 21 dpi.

As shown in Figure 4, the inactivated PRV HD/c vaccine conferred protection against the strain DX of PRV type II in a dose-dependent manner in mice. The survival rate of mice that received the HD/c vaccine (10^7.8^ TCID_50_) reached 90% after challenge with virulent DX strain at 21 dpi, and also reached 90% after challenge with virulent DX strain at 28 and 35 dpi, respectively. The protection rate also reached more than 70% in mice that were immunized by the HD/c vaccine with a titer of 10^7.3^ TCID_50_ and 10^6.8^ TCID_50_ after challenge at 21, 28, and 35 dpi. However, those mice that received the HD/c vaccine with a titer of 10^6.3^ TCID_50_ only had 50% survival rate when challenged with a virulent DX strain at 21 dpi (Figure 4A), 70% and 80% survival rate when challenged at 28 and 35 dpi, respectively (Figures 4A–C). On the contrary, most of mock-immunized mice died after the challenge of the virulent DX strain (Figures 4A–C). To further assess the protective efficacy of inactivated HD/c vaccine against the classical PRV, the immunized mice were challenged with PRV SC strain. The results showed that all HD/c-immunized mice survived from the challenge of classical PRV SC strain (Figure 4D). In contrast, only 10% of DMEM-vaccinated mice survived after challenge with the classical PRV SC strain and none of them survived for 5 days when challenged with the PRV DX strain (Figure 4D). These findings suggested that the inactivated HD/c vaccine had a good protection to PRV genotypes I and II.

### The Inactivated PRV HD/c Vaccine Confer Full Protection Against Lethal Challenge of PRV DX in Piglet

**Figure 5:**
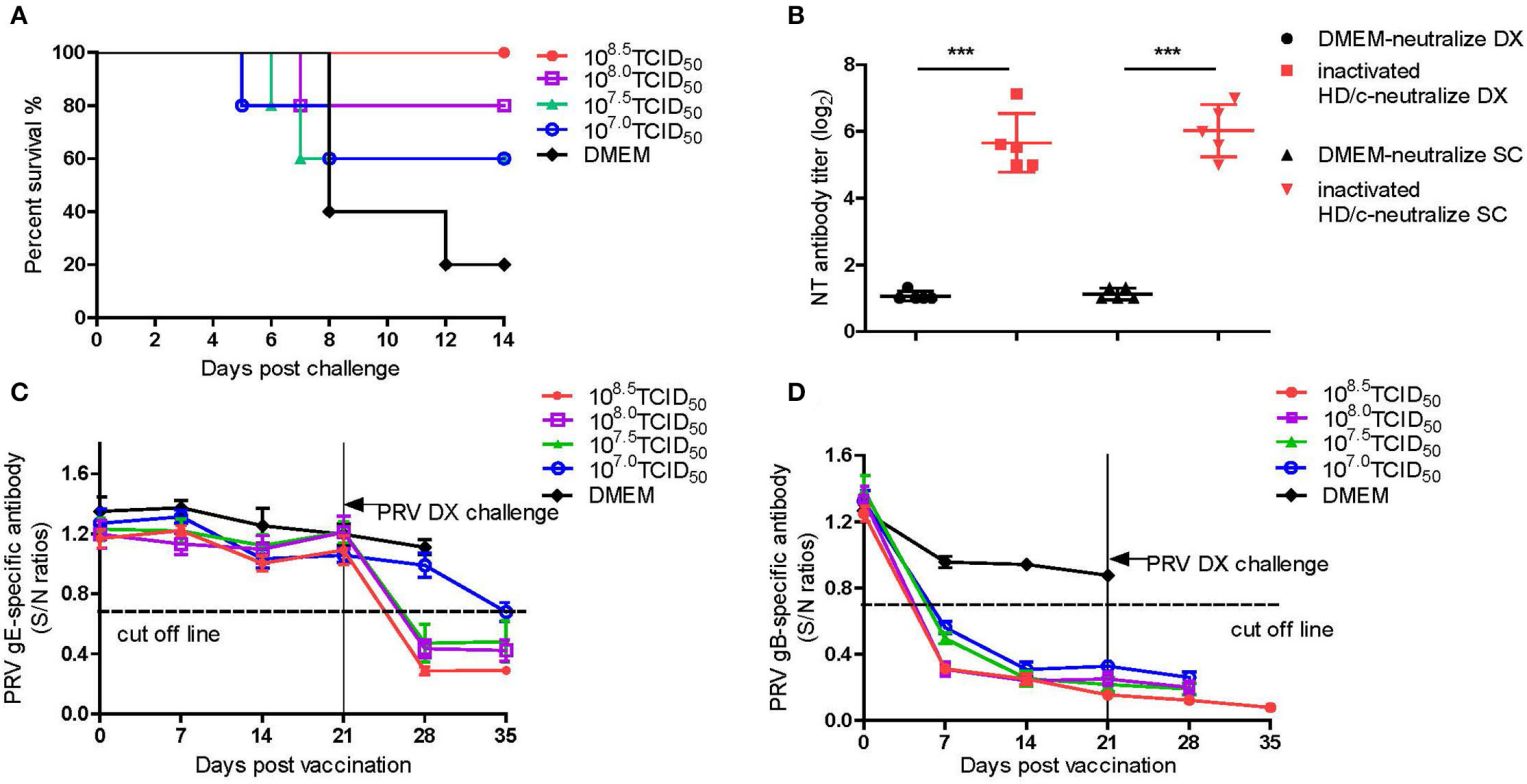
Protective efficacy of inactivated PRV HD/c immunization in pigs. **(A)** Survival percentage of piglets after challenge with DX strain. **(B)** The titers of neutralizing antibody against PRV DX or SC, ***p < 0.0001. **(C)** PRV gE-specific antibodies were detected with PRV gE Test Kit (IDEXX, USA) based on blocking ELISA. If S/N ratio < 0.6, the sample is positive for gE antibodies. **(D)** PRV gB-specific antibodies were detected with PRV gB Test Kit (IDEXX, USA) based on blocking ELISA. If S/N ratio < 0.6, the sample is positive for gB antibodies. Standard deviations were shown as error bars.

In order to assess the efficacy of inactivated PRV HD/c vaccine against the emerging PRV variant, 14-day-old piglets were immunized with different dose of inactivated PRV HD/c or DMEM, followed by challenge with the virulent PRV DX strain at 21 dpi. As shown in Figure 5, the protection of the HD/c vaccine against virulent PRV was also the dose-dependent in piglets. All piglets that were vaccinated with a dose of 10^8.5^ TCID_50_ inactivated HD/c survived and did not show any typical signs of PR after challenged with the DX virus (Figure 5A). When immunized at a dose of 10^8.0^ TCID_50, one_ out of five piglets displayed fever and depression symptoms from 5 dpc and died at 7 dpc after challenge (Figure 5A). When immunized with a dose of 10^7.0^ or 10^7.5^ TCID_50_ inactivated PRV HD/c, the protection rate of piglets decreased to 60% (Figure 5A). However, all the DMEM-injected piglets showed typical PR symptoms, including fever, depression, anorexia, belly breathing, tremor, and opisthotonos from 4 dpc, and the mortality rate reached 80% at 12 dpc (Figure 5A).

The titers of antibodies against gB and gE as well as the neutralization antibodies (NT) against the DX and SC viruses were measured for the piglets that received 10^8.5^ TCID_50_ of inactivated PRV HD/c. The results showed that the piglets immunized with a dose of 10^8.5^ TCID_50_ provided very significant DX- and SC-specific NT responses (*p* < 0.0001) in comparison with the mock-immunized piglets at 21 dpi (Figure 5B). The PRV gE antibodies were not detectable in the piglets before challenge (Figure 5C). However, after challenge, all inoculated piglets seroconverted for gE antibody at 7 dpc except that the piglets received a dose of 10^7.0^TCID_50_ HD/c seroconverted at 14 dpc (Figure 5C). All the vaccinated piglets seroconverted rapidly at 7 dpi for gB antibody and the antibody titers increased progressively before challenge with PRV DX strain (Figure 5D). In contrast, the gB and gE antibodies were not detected in DMEM-vaccinated group (Figures 5C,D).

**Figure 6:**
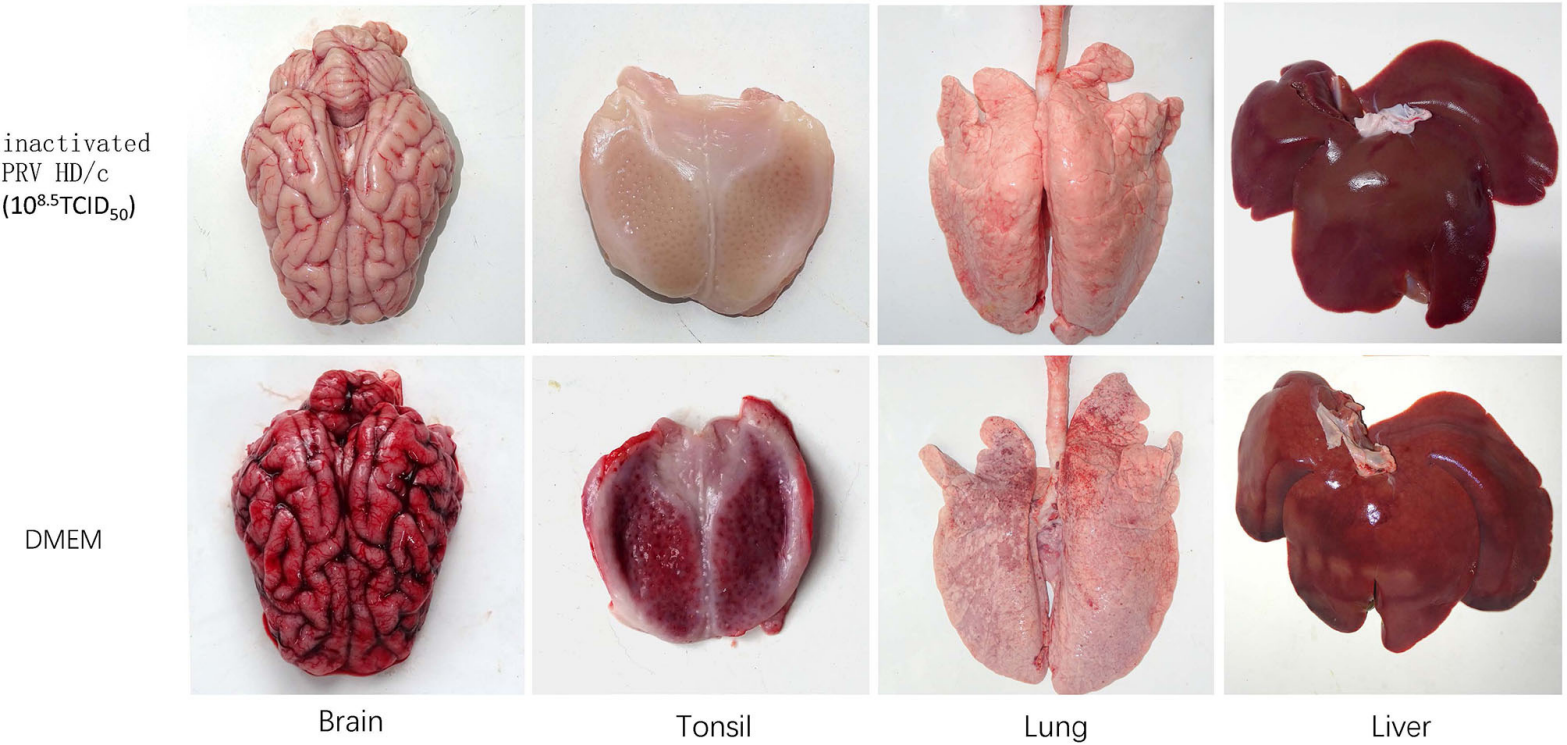
Pathological changes of inactivated piglets challenged with the PRV strain DX. Groups of pigs (*n* = 5 per group) were vaccinated with inactivated PRV HD/c (10^8.5^TCID_50_) or DMEM, and challenged with the PRV strain DX (10^5.4^TCID_50_) at 21 dpi. The brain, tonsil, lung, and liver of inactivated piglets were collected and subjected to pathological examinations.

**Figure 7:**
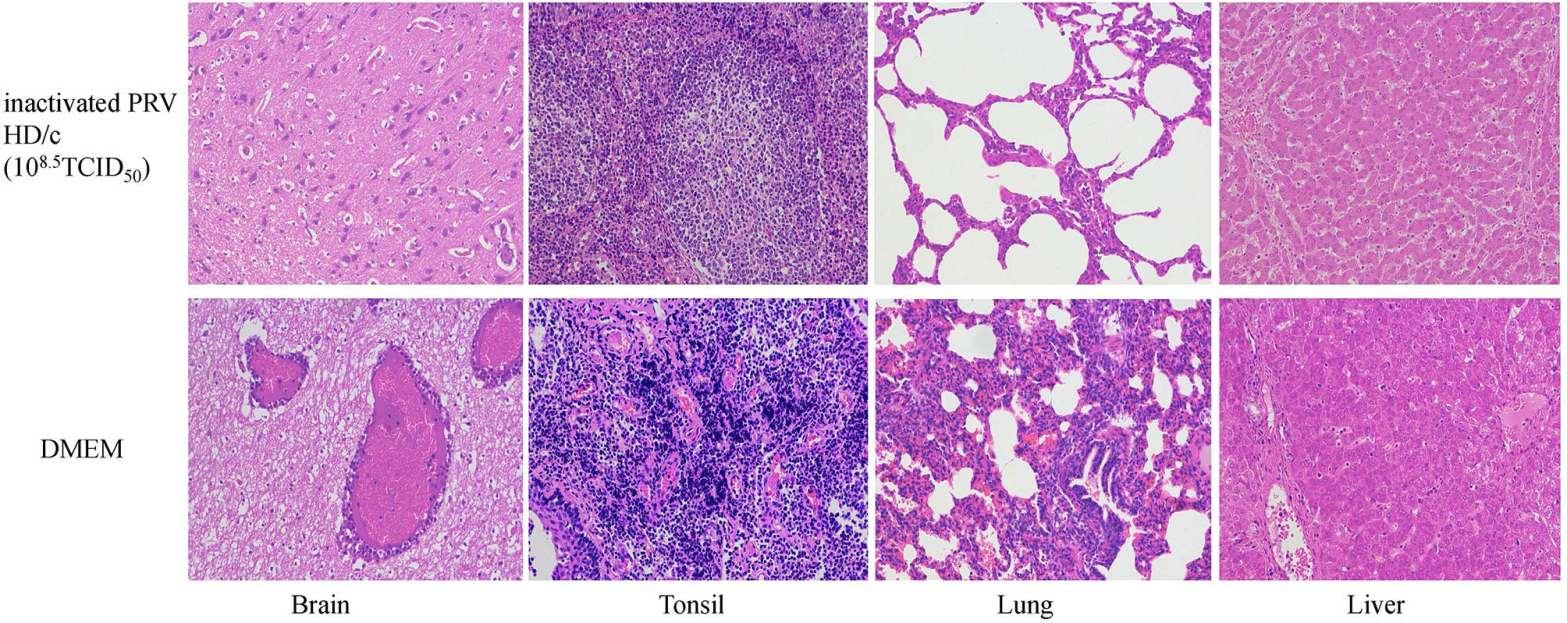
Histopathological observation of vaccinated piglets challenged with the PRV strain DX. Groups of pigs (*n* = 5 per group) were vaccinated with inactivated PRV HD/c (10^8.5^TCID_50_) or DMEM, and challenged with the strain DX (10^5.4^TCID_50_) at 21 dpi. Histopathological changes were observed in brain, tonsil, lung and liver of piglets. Sections were stained with hematoxylin and eosin (HE) and photographed at 200 × magnification.

Histopathological examination showed that after challenge with the virulent DX virus, no pathological lesions were observed in the tested tissues of the immunized piglets, whereas the mock-immunized piglets showed serious cerebral and tonsil hyperemia, many pinpoint hemorrhages in the lungs, and multiple small focal areas of necrosis in spleen and liver (Figure 6). In addition, infiltration of inflammatory cells and serious perivascular cuffing in the cerebrum, interstitial widening and inflammatory cell infiltration in the lung, necrosis in the tonsil were also observed in the mock-immunized group whereas lesions in HD/c-immunized piglets with challenge of the DX virus were much milder than the DMEM group, and no apparent histopathological changes were observed in the immunized piglets that did not infected with the DX virus (Figure 7).

### Protective Efficacy of Maternal Antibodies

**Figure 8:**
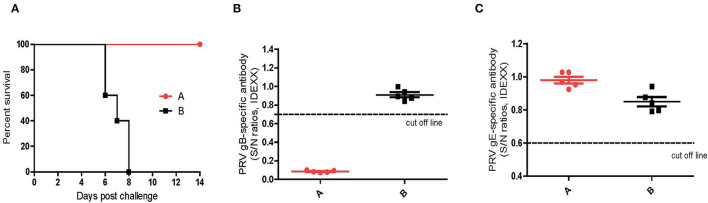
Protective efficacy of maternal antibodies. **(A)** Survival percentage of piglets after challenge with PRV strain DX. **(B)** PRV gB-specific antibodies were detected with PRV gB Test Kit (IDEXX, USA) at 0 dpc. If S/N ratio < 0.6, the sample is positive for gB antibodies. **(C)** PRV gE-specific antibodies were detected with PDRV gE Test Kit (IDEXX, USA) at 0 dpc. If S/N ratio < 0.6, the sample is positive for gE antibodies. Standard deviations were shown as error bars.

As shown in Figure 8, the newborn piglets farrowed by the vaccinated sows (Group A) survived from the challenge of the virulent DX virus and did not show any typical PR signs, whereas all the piglets farrowed by the un-vaccinated sows (group B) succumbed at 8 dpi. Serological tests showed that the newborn piglets in group A was positive for PRV gB antibodies and negative for gE antibodies at 4 weeks of age, while both the gB and gE antibodies were not detectable in piglets in group B (Figures 8B,C). These results suggested that the maternal antibodies induced by the inactivated HD/c vaccine is protective to the newborn piglet.

## Discussion

The emergence of PRV variants (genotype II) caused substantial economic losses to pig industry in China since later of 2011 (Yao et al., 2021). The strategies based on the differentiating infected from vaccinated individuals (DIVAs) vaccination program is the best way to eradicate PR in commercial swine populations (van Oirschot, 1999). In this study, we constructed a novel recombinant PRV with gE/TK deletion (PRV HD/c) by Cre/loxp system and made an inactivated PRV HD/c vaccine. When immunized with a dose of 10^8.5^ TCID_50_/ml in piglets of inactivated HD/c antigen, complete protection was achieved against the lethal challenge of parental PRV DX strain and associated with the high titers of neutralizing antibody against the PRV DX and SC strain. When immunized with a dose of 10^7.8^ TCID_50_/ml in mice of inactivated HD/c antigen, strong protection was achieved against the lethal challenge of parental PRV DX strain.

By the genomic comparison of the newly-emerged PRV variants and the classical PRV strain, multiple antigenic shifts were found in the new PRV strains (Yu et al., 2014). Several reports showed that PRV variants were more virulent and lethal than the classical strains in some animal experiments of mice, pigs, and sheep. Moreover, the Bartha-K61 strain vaccine cannot provide effective protection against PRV variants (An et al., 2013; Hu et al., 2015; Yu et al., 2016; He et al., 2019). In the past decade, many different recombinant viruses based on the emerging PRV strain were constructed and evaluated for their safety and immunogenicity in mice and piglets. Some live-attenuated vaccines and inactivated vaccines also can provide protection against the parental strain in mice and piglets (Wang et al., 2018; Li et al., 2020, 2021; Lv et al., 2021). However, there are few reports on the evaluation of the safety and immunogenicity of an inactivated vaccine in pregnant sows. Our study showed that the PRV HD/c inactivate vaccine can not only protect the pregnant sow completely but also provide stronger maternal antibodies against the challenge of the virulent PRV DX strain, and the newborn piglets from prior immunized sows was 100% protected against the challenge of the virulent PRV. These results suggested that the PRV HD/c inactivated vaccine may provide broad protection against the epidemic PRV strains in China.

Pseudorabies virus has a wide of host range (Lin et al., 2019) and live-attenuated vaccine strain was shown to cause latent infection in the brains, the use of live-attenuated vaccine may have potential safety issues in the pig industry in a long-term run. PRV can be divided into two main clades with frequent interclade and intraclade recombinations. The PRV type II variants are currently the most prevalent genotype worldwide, and commonly involved in cross-species transmission including humans (Lu et al., 2021). PRV may be still circulating in swine herds and a risk related to cross-species transmission in China (He et al., 2019). The commercial live-attenuated vaccine against PRV is a hidden risk for dogs and their owners (Lin et al., 2019). The use of inactivated vaccine based on the gE/TK-deleted HD/c strain in this study may be a good option to reduce the co-presence of live-attenuated vaccine and wide-type PRVs.

## Conclusions

We constructed a *gE*/*TK* genes-deficient PRV based on a PRV genotype II variant DX strain and developed an inactivated vaccine. This vaccine exhibited good safety, immunogenicity, and protection in the mice and piglets against the classical or emerging PRV strains and conferred maternal protection. Our results indicated that the inactivated PRV HD/c vaccine may be a safe and effective PRV vaccine candidate for the eradication of PR outbreaks in China.

## Data Availability Statement

The original contributions presented in the study are included in the article/Supplementary Material, further inquiries can be directed to the corresponding author/s.

## Ethics Statement

The animal study was reviewed and approved by Animal Research Committee Guidelines of Zhejiang University (No. ZJU20170066).

## Author contributions

J-YZ: conceptualization. Y-LJ and DY: methodology and writing paper manuscripts. GX and X-HQ: animal experiment. Y-MH, C-MF, and C-FF: sequencing and construction of evolutionary tree. W-RD, YY, and J-YG: writing review and editing. All authors have read and agreed to the published version of the manuscript.

## Funding

This work was supported by the Key Research & Development Program of the Zhejiang Province (Grant No. 2020C02011) and the National Key R&D program of China (Grant No. 2016YFD0500102).

## Conflict of Interest

The authors declare that the research was conducted in the absence of any commercial or financial relationships that could be construed as a potential conflict of interest.

## Publisher's Note

All claims expressed in this article are solely those of the authors and do not necessarily represent those of their affiliated organizations, or those of the publisher, the editors and the reviewers. Any product that may be evaluated in this article, or claim that may be made by its manufacturer, is not guaranteed or endorsed by the publisher.

## References

[B1] An T.-Q. Peng J.-M. Tian Z.-J. Zhao H.-Y. Li N. Liu Y.-M.. (2013). Pseudorabies virus variant in Bartha-K61-vaccinated pigs, China, 2012. Emerg. Infect. Dis. 19, 1749–1755. 10.3201/eid1911.130177 24188614 PMC3837674

[B2] Boadella M. Gortázar C. Vicente J. Ruiz-Fons F. (2012). Wild Boar: an increasing concern for Aujeszky's disease control in pigs? BMC Vet. Res. 8, 7. 10.1186/1746-6148-8-7 22251441 PMC3274458

[B3] Cong X. Lei J.-L. Xia S.-L. Wang Y.-M. Li Y. Li S.. (2016). Pathogenicity and immunogenicity of a GE/GI/TK gene-deleted Pseudorabies virus variant in susceptible animals. Vet. Microbiol. 182, 170–177. 10.1016/j.vetmic.2015.11.022 26711045

[B4] Dong B. Zarlenga D. S. Ren X. (2014). An overview of live attenuated recombinant pseudorabies viruses for use as novel vaccines. J. Immunol. Res. 2014, 824630. 10.1155/2014/824630 24995348 PMC4068083

[B5] Ferrari M. Gualandi G. L. Corradi A. Monaci C. Romanelli M. G. Tosi G.. (1998). Experimental infection of pigs with a thymidine kinase negative strain of Pseudorabies virus. Comp. Immunol. Microbiol. Infect. Dis. 21, 291–303. 10.1016/s0147-9571(98)00012-5 9775359

[B6] Fonseca A. A. Camargos M. F. de Oliveira A. M. Ciacci-Zanella J. R. Patrício M. A. C. Braga A. C.. (2010). Molecular epidemiology of Brazilian Pseudorabies viral isolates. Vet. Microbiol. 141, 238–245. 10.1016/j.vetmic.2009.09.018 19828266

[B7] Gu Z. Dong J. Wang J. Hou C. Sun H. Yang W.. (2015). A novel inactivated GE/GI deleted pseudorabies virus (PRV) vaccine completely protects pigs from an emerged variant PRV challenge. Virus Res. 195, 57–63. 10.1016/j.virusres.2014.09.003 25240533

[B8] Hanson R. P.. (1954). The history of Pseudorabies in the United States. J. Am. Vet. Med. Assoc. 124, 259–261. 13142964

[B9] He W. Auclert L. Z. Zhai X. Wong G. Zhang C. Zhu H.. (2019). Interspecies transmission, genetic diversity, and evolutionary dynamics of Pseudorabies virus. J. Infect. Dis. 219, 1705–1715. 10.1093/infdis/jiy731 30590733

[B10] Hu R.-M. Zhou Q. Song W.-B. Sun E.-C. Zhang M.-M. He Q.-G.. (2015). Novel Pseudorabies virus variant with defects in TK, GE and GI protects growing pigs against lethal challenge. Vaccine 33, 5733–5740. 10.1016/j.vaccine.2015.09.066 26428456

[B11] Klupp B. G. Hengartner C. J. Mettenleiter T. C. Enquist L. W. (2004). Complete, annotated sequence of the Pseudorabies virus genome. J. Virol. 78, 424–440. 10.1128/jvi.78.1.424-440.2004 14671123 PMC303424

[B12] Kozlov A. M. Darriba D. Flouri T. Morel B. Stamatakis A. (2019). RAxML-NG: a fast, scalable and user-friendly tool for maximum likelihood phylogenetic inference. Bioinforma. Oxf. Engl. 35, 4453–4455. 10.1093/bioinformatics/btz305 31070718 PMC6821337

[B13] Li J. Fang K. Rong Z. Li X. Ren X. Ma H.. (2020). Comparison of GE/GI- and TK/GE/GI-gene-deleted Pseudorabies virus vaccines mediated by CRISPR/Cas9 and Cre/Lox systems. Viruses 12, E369. 10.3390/v12040369 32230737 PMC7232343

[B14] Li W. Zhuang D. Li H. Zhao M. Zhu E. Xie B.. (2021). Recombinant Pseudorabies virus with GI/GE deletion generated by overlapping polymerase chain reaction and homologous recombination technology induces protection against the PRV variant PRV-GD2013. BMC Vet. Res. 17, 164. 10.1186/s12917-021-02861-6 33853597 PMC8048318

[B15] Lin W. Shao Y. Tan C. Shen Y. Zhang X. Xiao J.. (2019). Commercial vaccine against Pseudorabies virus: a hidden health risk for dogs. Vet. Microbiol. 233, 102–112. 10.1016/j.vetmic.2019.04.031 31176394

[B16] Lu J.-J. Yuan W.-Z. Zhu Y.-P. Hou S.-H. Wang X.-J. (2021). Latent Pseudorabies virus infection in medulla oblongata from quarantined pigs. Transbound. Emerg. Dis. 68, 543–551. 10.1111/tbed.13712 32615031

[B17] Lv L. Liu X. Jiang C. Wang X. Cao M. Bai J.. (2021). Pathogenicity and immunogenicity of a GI/GE/TK/UL13-gene-deleted variant Pseudorabies virus strain in Swine. Vet. Microbiol. 258, 109104. 10.1016/j.vetmic.2021.109104 34004569

[B18] Mettenleiter T. C.. (2008). Pseudorabies virus. Encycl. Virol. 341–351. 10.1128/jvi.75.19.8927-8936.2001

[B19] Mettenleiter T. C. Klupp B. G. Weiland F. Visser N. (1994). Characterization of a quadruple glycoprotein-deleted Pseudorabies virus mutant for use as a biologically safe live virus vaccine. J. Gen. Virol. 75(Pt 7), 1723–1733. 10.1099/0022-1317-75-7-1723 8021601

[B20] Minh B. Q. Schmidt H. A. Chernomor O. Schrempf D. Woodhams M. D. von Haeseler A.. (2020). IQ-TREE 2: new models and efficient methods for phylogenetic inference in the genomic era. Mol. Biol. Evol. 37, 1530–1534. 10.1093/molbev/msaa015 32011700 PMC7182206

[B21] Müller T. Hahn E. C. Tottewitz F. Kramer M. Klupp B. G. Mettenleiter T. C.. (2011). Pseudorabies virus in wild swine: a global perspective. Arch. Virol. 156, 1691–1705. 10.1007/s00705-011-1080-2 21837416

[B22] Pomeranz L. E. Reynolds A. E. Hengartner C. J. (2005). Molecular biology of Pseudorabies virus: impact on neurovirology and veterinary medicine. Microbiol. Mol. Biol. Rev. 69, 462–500. 10.1128/MMBR.69.3.462-500.2005 16148307 PMC1197806

[B23] Sun Y. Luo Y. Wang C.-H. Yuan J. Li N. Song K.. (2016). Control of swine Pseudorabies in China: opportunities and limitations. Vet. Microbiol. 183, 119–124. 10.1016/j.vetmic.2015.12.008 26790944

[B24] Szpara M. L. Tafuri Y. R. Enquist L. W. (2011). Preparation of viral DNA from nucleocapsids. J. Vis. Exp. 16, 3151. 10.3791/3151 21876519 PMC3217642

[B25] van Oirschot J. T.. (1999). Diva vaccines that reduce virus transmission. J. Biotechnol. 73, 195–205. 10.1016/s0168-1656(99)00121-2 10486928

[B26] van Oirschot J. T. Kaashoek M. J. Rijsewijk F. A. Stegeman J. A. (1996). The use of marker vaccines in eradication of herpesviruses. J. Biotechnol. 44, 75–81. 10.1016/0168-1656(95)00129-8 8717389

[B27] Wang C.-H. Yuan J. Qin H.-Y. Luo Y. Cong X. Li Y.. (2014). A novel GE-deleted Pseudorabies virus (PRV) provides rapid and complete protection from lethal challenge with the PRV variant emerging in Bartha-K61-vaccinated swine population in China. Vaccine 32, 3379–3385. 10.1016/j.vaccine.2014.04.035 24793946

[B28] Wang G.-S. Du Y. Wu J.-Q. Tian F.-L. Yu X.-J. Wang J.-B. (2018). Vaccine resistant Pseudorabies virus causes mink infection in China. BMC Vet. Res. 14, 20. 10.1186/s12917-018-1334-2 29351775 PMC5775606

[B29] Wang J. Song Z. Ge A. Guo R. Qiao Y. Xu M.. (2018). Safety and immunogenicity of an attenuated Chinese Pseudorabies variant by dual deletion of TKandgE genes. BMC Vet. Res. 14, 287. 10.1186/s12917-018-1536-7 30241529 PMC6150974

[B30] Wang T. Xiao Y. Yang Q. Wang Y. Sun Z. Zhang C.. (2015). Construction of a GE-deleted Pseudorabies virus and its efficacy to the new-emerging variant PRV challenge in the form of killed vaccine. Biomed. Res. Int. 2015, 684945. 10.1155/2015/684945 26457302 PMC4589612

[B31] Wang Y. Qiao S. Li X. Xie W. Guo J. Li Q.. (2015). Molecular epidemiology of outbreak-associated Pseudorabies virus (PRV) strains in central China. Virus Genes 50, 401–409. 10.1007/s11262-015-1190-0 25860998

[B32] Yao L. Hu Q. Chen S. Zhou T. Yu X. Ma H.. (2021). Recombinant Pseudorabies virus with TK/GE gene deletion and Flt3L co-expression enhances the innate and adaptive immune response via activating dendritic cells. Viruses 13, 691. 10.3390/v13040691 33923590 PMC8072707

[B33] Ye C. Zhang Q.-Z. Tian Z.-J. Zheng H. Zhao K. Liu F.. (2015). Genomic characterization of emergent Pseudorabies virus in China reveals marked sequence divergence: evidence for the existence of two major genotypes. Virology 483, 32–43. 10.1016/j.virol.2015.04.013 25965793

[B34] Yu T. Chen F. Ku X. Fan J. Zhu Y. Ma H.. (2016). Growth characteristics and complete genomic sequence analysis of a novel Pseudorabies virus in China. Virus Genes 52, 474–483. 10.1007/s11262-016-1324-z 27012685

[B35] Yu X. Zhou Z. Hu D. Zhang Q. Han T. Li X.. (2014). Pathogenic Pseudorabies virus, China, 2012. Emerg. Infect. Dis. 20, 102–104. 10.3201/eid2001.130531 24377462 PMC3884716

[B36] Yuan Q. Z. Wu Y. X. Li Y. X. Li Z. R. Nan X. (1983). The Pseudorabies vaccination research. I: Pseudorabies attenuated vaccine research. Chin. J. Prev. Vet. Med. 1, 1–16.

