# Supplementary material for: The Inactivated gE/TK Gene-Deleted Vaccine Against Pseudorabies Virus Type II Confers Effective Protection in Mice and Pigs
Source: Front Microbiol. 2022 Aug 4;13:943707. doi: 10.3389/fmicb.2022.943707 (PMC9389536; doi:10.3389/fmicb.2022.943707)
Supplement: Supplementary file 1 [file Data_Sheet_1.PDF]

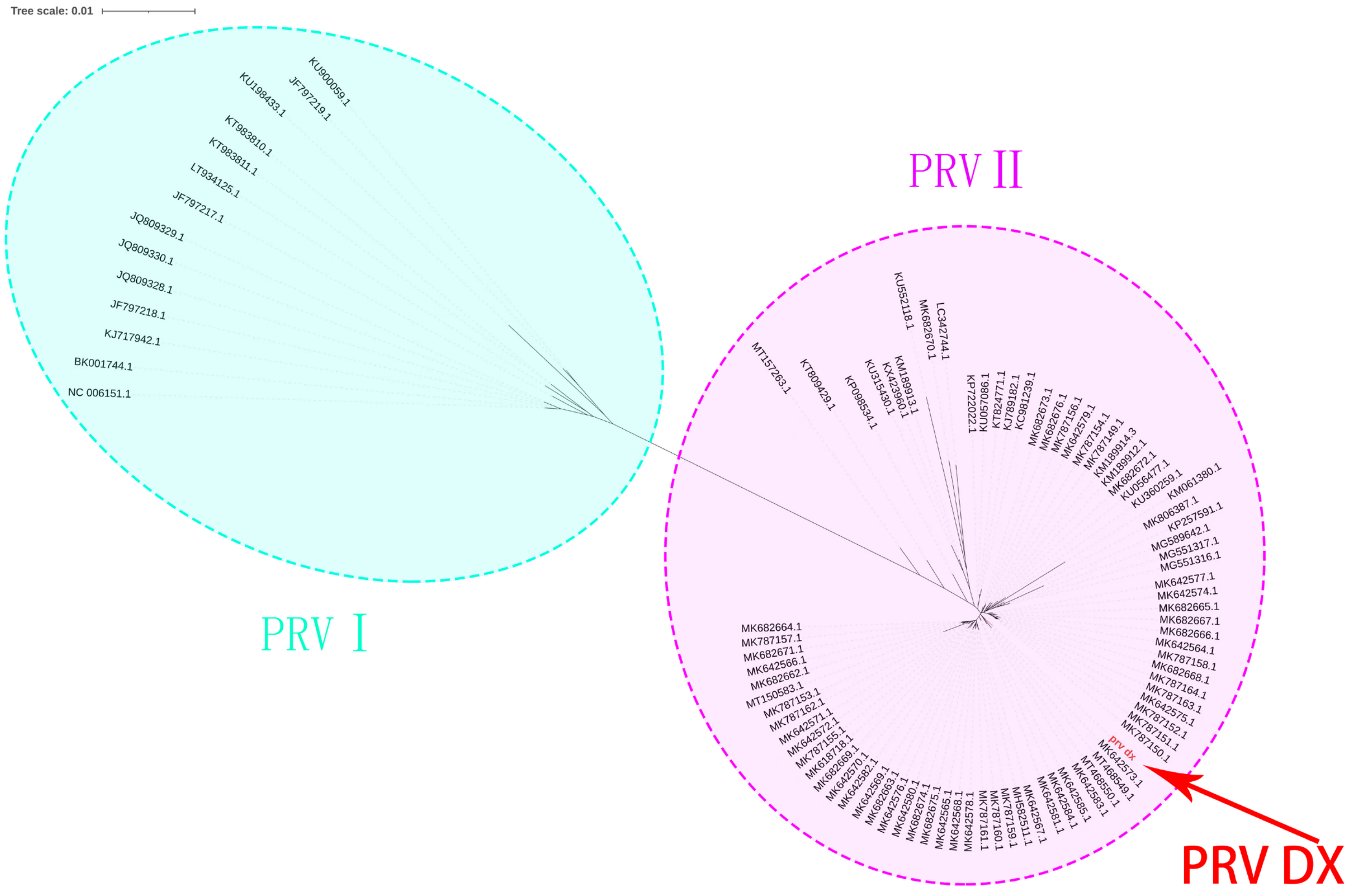

Supplemental Fig. 1. rootless phylogenetic tree.
